# Dental Engines

**Published:** 1874-06

**Authors:** 


### DENTAL ENGINES.

We are not yet in the possession of full knowledge, in reference to the present status of the Dental Engine *muddle;* but we ar· warranted in saying that it has passed that point, where the dentists need entertain no further apprehension as to infringements, prosecutions, litigations, etc. It is too often the case that dentists are frightened out of their wits by threats of prosecution for infringements of some patent. There is nothing that will cause them to cry out, *what shall we do to be saved*, like such threats.

We trust this little tempest will calm down now. Johnston & Bros. have come to the rescue with an improved engine, superior to the old one, as it is claimed, and have stepped outside of any other patents, that claimed to be infringed by the former engine.

We have an intimation that there is to be a new and valuable engine out soon from another quarter, that may make a strong competition with the Morrison engine.

We rejoice in, and welcome all the good and valuable things that may be brought forth.

